# Description of a new species of *Parotocinclus* (Siluriformes, Hypoptopomatinae) from the rio Tapajós basin

**DOI:** 10.3897/zookeys.634.9917

**Published:** 2016-11-21

**Authors:** Fábio F. Roxo, Gabriel S. C. Silva, Claudio Oliveira

**Affiliations:** 1 Universidade Estadual Paulista, Departamento de Morfologia, Laboratório de Biologia e Genética de Peixes, R. Prof. Dr. Antônio Celso Wagner Zanin, 250, Rubião Júnior, 18618–689, Botucatu, São Paulo State, Brazil

**Keywords:** Biodiversity, Cascudinhos, freshwater, Neotropical fish, taxonomy

## Abstract

A new species of *Parotocinclus* is described from three small tributaries of the rio Tapajós basin, Mato Grosso State, Brazil. The new species can be distinguished from its congeners by presenting the following characters: (1) a triangular dark blotch at the anterior base of the dorsal fin, (2) the absence of an adipose fin but presence of one small platelet at typical adipose-fin region, (3) the abdomen completely covered by dermal plates, (4) a pectoral girdle totally exposed, (5) a single series of bicuspid teeth, and (6) the higher number of bicuspid premaxillary and dentary teeth.

## Introduction

The subfamily Hypoptopomatinae currently includes the tribes Otothyrini, Neoplecostomini, and Hypoptopomatini (*sensu* Lujan et al. 2015) and is one of the most diversified and widespread groups of the Neotropical family Loricariidae, with about 210 species according to Eschmeyer and Fong (2016). Within Otothyrini, the genus *Parotocinclus* initially described by Eigenmann and Eigenmann (1889) as a subgenus of *Hisonotus* Eigenmann & Eigenmann, 1889 to include the species *Otocinclus maculicauda* Steindachner, 1887, presents 28 species (Eschmeyer 2016).

Recently, as a result of molecular studies, the genus *Parotocinclus* was recognized as non-monophyletic with several species more related to species assigned to other Otothyrini genera, mainly *Hisonotus*, than to the type species *Parotocinclus maculicauda* (e.g. Cramer et al. 2011; Roxo et al. 2014; Silva et al. 2016). This genus is distributed through almost all hydrographic systems in South America from the Guyana Shield drainages and Amazon Shield tributaries to the coastal drainages of eastern and southeastern Brazil, including the rio São Francisco basin (Sarmento-Soares et al. 2009; Lehmann et al. 2014, 2015).

Lehmann et al. (2014, 2015) proposed that several species included in *Parotocinclus*, namely: *Parotocinclus amazonensis* Garavello, 1977; *Parotocinclus britskii* Boeseman, 1974; *Parotocinclus collinsae* Schmidt & Ferraris, 1985; *Parotocinclus eppleyi* Schaefer & Provenzano, 1993; *Parotocinclus halbothi* Lehmann, Lazzarotto & Reis, 2014; *Parotocinclus longirostris* Garavello, 1988; *Parotocinclus polyochrus* Schaefer, 1988; and *Parotocinclus variola* Lehmann, Schvambach & Reis, 2015, could be part of a new genus. These authors suggested that this possible new genus could be diagnosed by (1) the presence of a triangular dark blotch at the anterior base of the dorsal fin; (2) a canal cheek plate on the ventral surface of head elongated posteriorly and contacting the cleithrum; and (3) the head and snout being elongated and with a *Y*-shaped, white or cream colored mark dorsally.

Here, we recognized a new Otothyrini species as a result of collection efforts in the rio Tapajós basin. The new species is described below in the genus *Parotocinclus*, but we will not be surprised if this new entity is reallocated into a new genus in a close future.

## Material and Methods

Measurements and counts were taken from the left side. The measurements followed Boeseman (1968) with modifications suggested by Armbruster and Page (1996), except for the folded dorsal-fin length. Furthermore, the following measurements were added: anal-fin spine length, lower caudal spine length, suborbital depth and mandibular ramus. Meristics followed Carvalho and Reis (2009) and Schaefer (1997). All measurements were taken point to point with digital calipers to the nearest 0.1 mm. All samples analyzed are deposited at the LBP – Laboratório de Biologia e Genética de Peixes, São Paulo State, Brazil; and MZUSP – Museu de Zoologia da Universidade de São Paulo, São Paulo State, Brazil. Abbreviations used throughout the text followed Carvalho and Reis (2009). One specimen of the new species was cleaned and double-stained (c&s) according to the method of Taylor and Van Dyke (1985).

## Results

### Parotocinclus dani sp. n.

Taxon classificationAnimaliaSiluriformesLoricariidae

http://zoobank.org/637C26FF-4E1D-4DA6-A810-F41FCEB1C976

Fig. 1 , Table 1

#### Holotype.

MZUSP 120737, 27.3 mm SL, municipality of Peixoto de Azevedo, Mato Grosso State, small tributary of rio Peixoto de Azevedo, drainage of rio Teles Pires, rio Tapajós basin, 10°23'10"S, 54°18'22"W, 18 August 2007, coll. JLO Birindelli, AL Netto-Ferreira & LM Souza.

#### Paratypes.

All from Brazil, Mato Grosso State, rio Tapajós basin. MZUSP 96785, 126, 17.8–26.7 mm SL, collected with holotype. LBP 22089, 1, 26.9 mm SL, 1 c&s, 27.3 mm SL, collected with holotype. MZUSP 96194, 18, 16.7–24.7 mm SL, municipality of Paranaíta, rio Teles Pires, 09°27'31"S, 56°29'19"W, 30 September 2007, coll. LM Souza, AL Netto-Ferreira. MZUSP 96225, 5, 17.3–24.1 mm SL, municipality of Paranaíta, rio Teles Pires, 09°25'44"S, 56°32'36"W, 29 September 2007, coll. LM Souza, AL Netto-Ferreira.

#### Diagnosis.

The new species *Parotocinclus dani* can be distinguished from all congeners, except *Parotocinclus amazonensis*, *Parotocinclus bidentatus*, *Parotocinclus britskii*, *Parotocinclus eppleyi*, *Parotocinclus longirostris*, *Parotocinclus polyochrus*, and *Parotocinclus variola* by one character proposed by Lehmann et al. (2014, 2015): the presence of a triangular dark blotch at the anterior base of the dorsal fin, Fig. 2a (*vs.* absence Fig. 2b). The new species can be distinguished from *Parotocinclus amazonensis*, *Parotocinclus britskii*, *Parotocinclus collinsae*, *Parotocinclus eppleyi*, *Parotocinclus halbothi*, *Parotocinclus longirostris*, *Parotocinclus polyochrus*, and *Parotocinclus variola* by the absence of an adipose fin but presence of one small platelet at typical adipose-fin region, Fig. 3 (*vs.* presence of a poorly developed to well-developed adipose fin); from *Parotocinclus bahiensis*, *Parotocinclus cearensis*, *Parotocinclus cesarpintoi*, *Parotocinclus jumbo*, *Parotocinclus prata*, *Parotocinclus robustus*, and *Parotocinclus spilosoma* by the abdomen completely covered by dermal plates (*vs.* abdomen totally exposed or with few small and dispersed platelets); from *Parotocinclus cearensis*, *Parotocinclus cesarpintoi*, *Parotocinclus jumbo*, *Parotocinclus prata*, *Parotocinclus robustus*, *Parotocinclus spilosoma*, and *Parotocinclus spilurus* by having the pectoral girdle totally exposed (*vs.* the pectoral girdle medially covered by skin and exposed only laterally); from *Parotocinclus bidentatus* by the presence of a single series of bicuspid teeth (*vs.* the presence of a series of unicuspid teeth behind the series of bicuspid teeth of the dentary and premaxilla), and by the higher number of bicuspid premaxillary teeth 15–25, mode 21 (*vs.* 6–12, mode 9) and bicuspid dentary teeth 15–22, mode 21 (*vs.* 4–10, mode 7).

**Figure 1.**
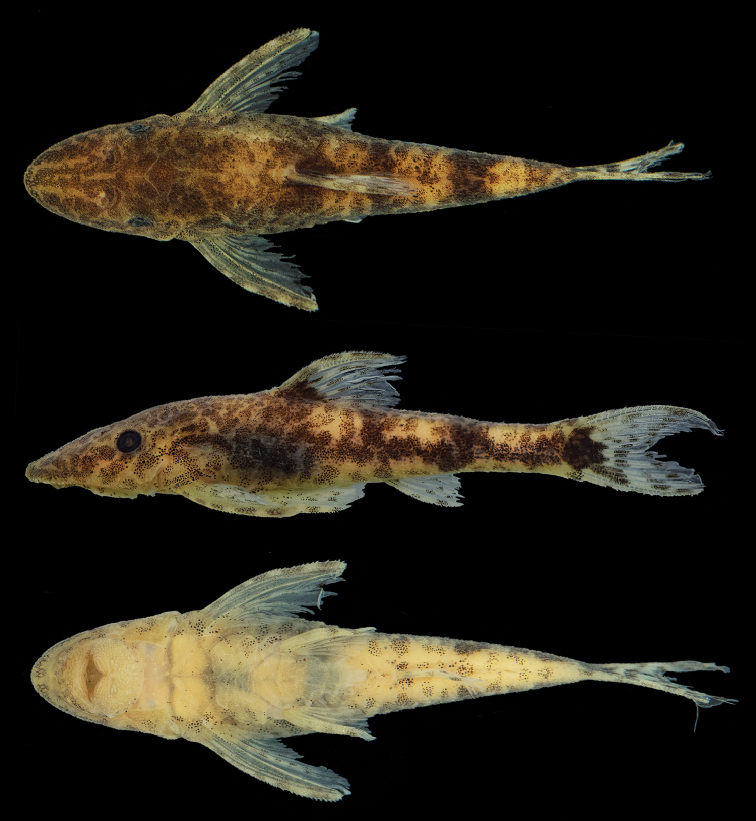
*Parotocinclus dani*, MZUSP 120737, 27.3 mm SL, holotype from small tributary of rio Peixoto de Azevedo, rio Tapajós basin, municipality of Peixoto de Azevedo, Mato Grosso State, Brazil.

**Figure 2.**
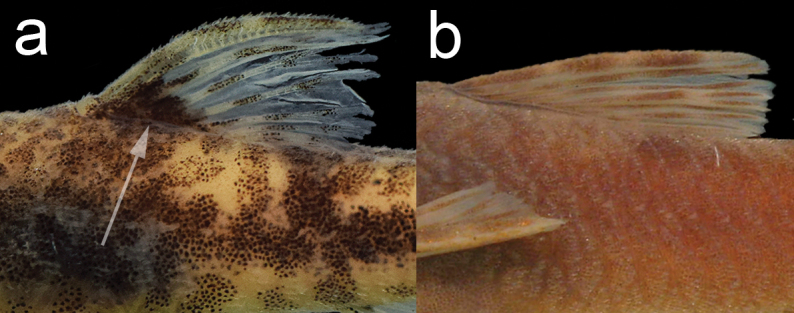
Photographs showing **a** the presence of a triangular dark blotch at the anterior base of the dorsal fin in *Parotocinclus dani* (arrow), holotype, MZUSP 120737, 27.3 mm SL; and **b** absence of the triangular dark blotch in *Parotocinclus prata*, holotype, MZUSP 68359, 38.2 mm SL. Photo: CH Zawadzki.

**Figure 3.**
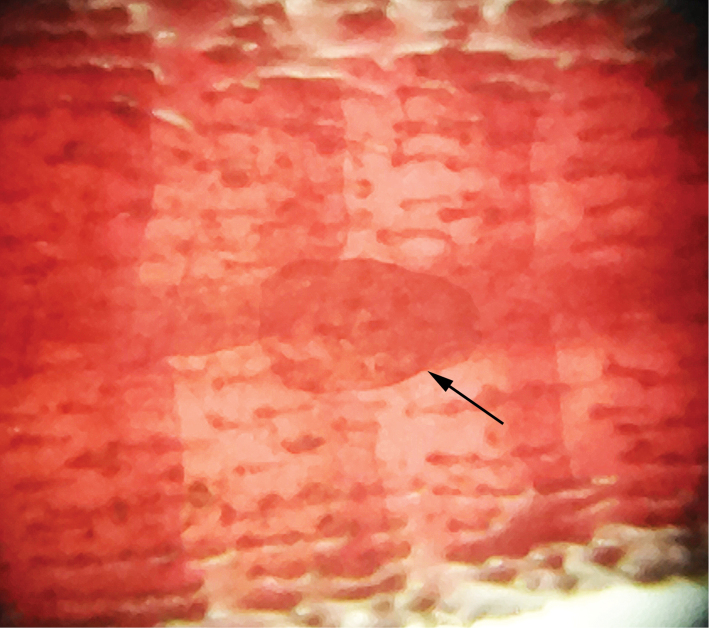
Picture showing (arrow) the single dorsal plate in the caudal peduncle at typical adipose-fin region of *Parotocinclus dani*, LBP 22089, 27.3 mm SL.

#### Description.

Morphometric and meristic data shown in Table 1. Small size Loricariidae, holotype 27.3 mm SL; paratypes 20.7–27.3 mm SL. Dorsal profile in lateral view straight from snout tip to anterior portion of parieto-supraoccipital, slightly convex to dorsal-fin origin. Dorsal-fin base straight, slightly concave and descending from posterior end of dorsal-fin base to caudal peduncle. Ventral profile in lateral view slightly concave from snout tip to anal-fin origin, slightly convex from anal-fin base to caudal-fin origin. In dorsal view body progressively narrowing posteriorly from cleithrum to caudal peduncle and anteriorly to snout tip. Greatest body depth at dorsal-fin origin. Cross-section of body between pectoral and pelvic fins dorsally rounded and ventrally flat; cross-section of caudal peduncle ellipsoid, round laterally, flat dorsally and ventrally.

**Table 1.** Morphometric and meristic data for *Parotocinclus dani* (N = 18).

	Holotype	Range	Mean	SD
**SL**	27.3	20.7–27.3	24.0	1.93
**Percent of SL**				
Predorsal length	48.8	45.9–50.1	47.7	1.24
Preanal length	66.2	63.3–67.1	65.6	1.07
Head length	38.0	36.4–46.9	39.1	2.26
Cleithral width	23.4	12.7–26.8	22.4	4.84
Dorsal-fin spine length	25.8	24.0–30.6	26.4	1.68
Base of dorsal fin length	12.2	12.2–15.1	14.0	0.86
Thorax length	17.5	11.6–21.3	14.4	2.16
Pectoral-fin spine length	28.6	27.1–32.8	29.8	1.47
Abdomen length	22.4	19.8–23.5	21.4	1.19
Pelvic-fin spine length	16.7	16.2–21.0	17.9	1.33
Postanal length	26.7	23.6–29.1	26.7	1.26
Caudal peduncle depth	8.4	8.4–9.4	8.8	0.29
Anal width	13.3	12.5–13.8	13.2	0.44
Snout-opercle length	28.0	20.0–30.8	26.8	4.29
Anal-fin spine length	16.2	15.1–18.8	17.0	1.04
Lower caudal spine length	29.7	22.1–30.3	27.1	2.41
**Percent of HL**				
Head width	62.3	48.0–65.7	59.8	3.91
Head depth	44.9	36.4–49.0	43.6	2.42
Snout length	53.3	43.0–55.6	51.6	2.69
Interorbital width	40.5	31.6–40.8	38.4	2.15
Orbital diameter	12.5	9.7–17.0	14.1	2.06
Suborbital depth	17.8	15.4–20.6	18.7	1.35
Mandibular ramus	10.5	6.9–11.2	9.2	1.40
**Meristics**				
Lateral plates	24	24–26	25	–
Premaxillary teeth	22	15–25	21	–
Dentary teeth	21	15–22	21	–

Top of head in parieto-supraoccipital region and between orbits convex; superior margin of orbits elevated. Eyes moderately small (9.7–17.0% of HL), and dorsolaterally positioned. Snout pointed and rounded in dorsal view. Nostril small. Body and almost all head plates covered with minute, uniformly sized and evenly distributed odontodes. Absence of tufts of hypertrophied odontodes at posterior medial portion of parieto- supraoccipital or crests on head. Dorsal and ventral anterior margin of snout covered with larger odontodes compared to rest of head. Lips moderately developed and rounded; lower lip far from reaching pectoral girdle. Papillae uniformly distributed on base of dentary and premaxilla, getting smaller distally. Lower lip larger than upper. Maxillary barbel present and poorly developed. Teeth slender and bicuspid; medial cusp larger than lateral cusp. Left premaxillary teeth 15–25 (mode 21). Left dentary teeth 15–22 (mode 21).

Dorsal fin ii,7; its origin slightly posterior to pelvic-fin origin; when depressed reaching beyond vertical line through anal-fin insertion. Tip of branched dorsal-fin rays reaching vertical line slightly posterior of anal-fin origin. Dorsal-fin spinelet *V*-shaped, laterally extended; dorsal-fin locking mechanism functional. Pectoral fin i,6; tip of longest pectoral-fin ray almost reaching vertical line through center of horizontal pelvic-fin length when depressed. Pectoral axial slit present between pectoral-fin base and lateral process of cleithrum. Lateral margin of pectoral spine possessing odontodes increasing in size posteriorly. Pelvic fin i,5; tip not exceeding anal-fin origin when depressed. Males with flap along dorsal margin of unbranched pelvic-fin ray, absent in females. Anal fin i,5; tip of unbranched anal-fin ray reaching 7th to 9th plate from anal-fin origin. Adipose-fin absent but with small unpaired plates in typical adipose fin region. Caudal fin i,14,i; distal margin forked. Lateral plate series formed by 24–26 (mode 25) plates. Lateral line with one or two unperforated plates in line of pores along mid length of body, terminating in two plates preceding last lateral plate. Abdomen completely covered by dermal plates. Cleithrum partly enclosed by ventral lamina of coracoids.

#### Color in alcohol.

Background color dark yellowish-brown in dorsal portion of body and yellowish tan in ventral portion. Dorsal surface of head dark brownish, except for striking *V*-shaped yellowish tan mark from rostral plate passing through nares to orbital margins. Irregular and conspicuous dark brownish longitudinal stripe along lateral line. Four dark brownish bars crossing dorsum, reaching longitudinal stripe on sides of trunk: first below dorsal-fin origin, second at end of dorsal-fin base, third at adipose fin region, and fourth more inconspicuous at end of caudal peduncle. Dorsal, pectoral, and pelvic fins with dark chromatophores, forming irregular sets of bands: five on dorsal and pectoral fins, three to four on pelvic-fin, and four on anal fin. Dorsal-fin with triangular dark blotch at anterior base. Unpaired plates in typical adipose-fin region yellowish tan. Caudal-fin hyaline, except for one black spot at its origin extending to ventral lobe, and two almost inconspicuous bands. Entire body covered with irregularly distributed chromatophores.

#### Sexual dimorphism.

Adult males can be distinguished from females by presenting two characters: (1) presence of a papilla at urogenital opening (*vs.* papilla absent in females), and (2) unbranched pelvic-fin ray supporting a dermal flap on proximal dorsal surface (*vs.* dermal flap absent in females).

#### Distribution.

**Figure 4.**
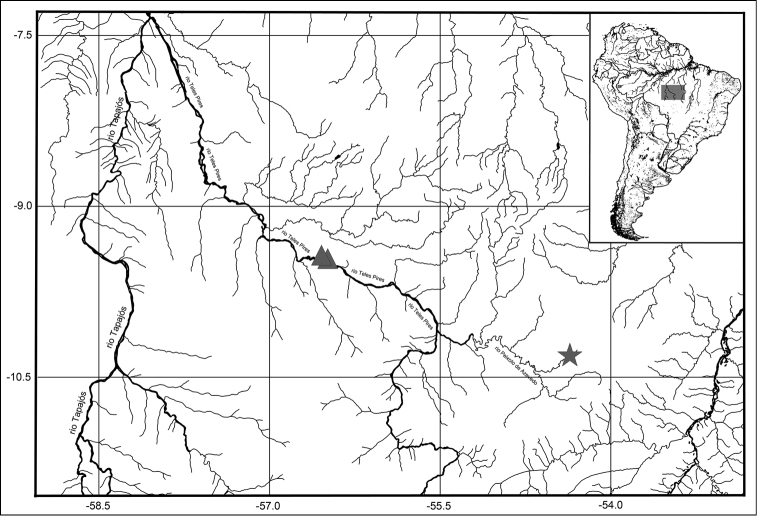
Map of the distribution of *Parotocinclus dani*. Star = holotype locality, affluent of the rio Peixoto de Azevedo. Triangles = paratype localities at the rio Teles Pies. All are drainages of the rio Tajajós basin, Mato Grosso State, Brazil.

The new species is known from three drainages of rio Tapajós in Mato Grosso State, Brazil (Fig. 4). Two from the rio Teles Pires, in the municipality of Paranaíta and from a small tributary of rio Peixoto de Azevedo, in the municipality of Peixoto de Azevedo.

#### Etymology.

The specific name “dani” is in honor of Daniela Fernandes Roxo, FF Roxo’s sister.

#### Discussion.

Lehmann et al. (2014, 2015) proposed that the species *Parotocinclus amazonensis*, *Parotocinclus britskii*, *Parotocinclus collinsae*, *Parotocinclus eppleyi*, *Parotocinclus halbothi*, *Parotocinclus longirostris*, *Parotocinclus polyochrus*, and *Parotocinclus variola* should be part of a new genus of Otothyrini based on the following synapomorphies (1) presence of a triangular dark blotch at the anterior base of the dorsal fin, (2) canal cheek plate on the ventral surface of the head elongated posteriorly and contacting the cleithrum, and (3) head and snout elongated and with a *Y*-shaped, white or cream colored mark dorsally. The first character is apparently conserved and may help to diagnose a new genus within Otothyrini. However, it is also present in *Parotocinclus bidentatus* (see the holotype picture in Gauger and Buckup 2005, Fig. 5). The second character is absent in *Parotocinclus dani* and the third character is present not only in *Parotocinclus dani* and species of this possible new genus proposed by Lehmann et al. (2014, 2015), but also in species of *Hisonotus* – e.g., *Hisonotus acuen* and *Hisonotus chromodontus*, species of *Curculionichthys* – e.g., *Curculionichthys luteofrenatus*, *Curculionichthys paresi*, and species of *Epactionotus* – e.g., *Epactionotus bilineatus*, *Epactionotus itaimbezinho* and *Epactionotus gracilis*. Given the above information, it is clear that new analyses are necessary to recognize this putative new genus more accurately.

Carvalho and Datovo (2012) described a new Otothyrini species, *Hisonotus bockmanni*, from small tributaries of the rio Teles Pires, drainages of the rio Tapajós. This species lacks an adipose fin as the new species *Parotocinclus dani*, and presents several small platelets at typical adipose-fin region. Furthermore, *Hisonotus bockmanni* shows a triangular dark blotch at the anterior base of the dorsal-fin suggesting that this species may also be part of the new genus proposed by Lehmann et al. (2014, 2015). However, we could not examine the clear and stained specimens of *Hisonotus bockmanni* to verify if this species presents the first character proposed by Lehmann et al. (2014, 2015), i.e., a canal cheek plate on the ventral surface of the head elongated posteriorly and contacting the cleithrum. *Hisonotus bockmanni* and the new species *Parotocinclus dani* could be part of the same monophyletic genus and may be closely related. Notwithstanding, *Hisonotus bockmanni* can be distinguished from its congeners by the presence of the following characters of coloration pattern proposed by Carvalho and Datovo (2012): (1) the snout with unpigmented, rostrocaudally elongate ellipse anterior to each naris; (2) the dark-brown pigmented pre-dorsal region with five unpigmented white spots arranged as an anteriorly chevron-shaped blotch with three spots anteriorly of dorsal-fin and two posterior spots lateral to and coequal with insertion of dorsal-fin spine; and (3) the caudal-fin lacking pigments on half of membrane and rays.

### Comparative material

*Corumbataia cuestae* Britski, 1997: LBP 3688, 3, 28.5–29.9 mm SL, municipality of Botucatu, São Paulo State, upper rio Paraná basin.

*Curculionichthys insperatus* (Britski & Garavello, 2003): LBP 1316, 4 (1 c&s), 23.9–27.7 mm SL, municipality of Botucatu, São Paulo State, rio Tietê basin; LBP 1344, 2, 22.9–24.9 mm SL, municipality of Botucatu, São Paulo State, rio Tietê basin.

*Hisonotus bocaiuva* Roxo, Silva, Oliveira & Zawadzki, 2013: MZUSP 112204, 24.2 mm SL, holotype, municipality of Bocaiúva, Minas Gerais State, rio São Francisco basin; LBP 9817, 9, 4 c&s, 18.3–23.2 mm SL, municipality of Bocaiúva, Minas Gerais State, rio São Francisco basin.

*Hisonotus bockmanni* Carvalho & Datovo, 2012: MZUSP 116430, 2, 16.3–18.5 mm SL, municipality of Paranaitá, Mato Grosso State, rio Tapajós basin.

*Hisonotus francirochai* (Ihering, 1928): LBP 5026, 1, 34.6 mm SL, municipality of Rio Claro, São Paulo State, rio Tietê basin.

*Hisonotus notatus* Eigenmann & Eigenmann, 1889: LBP 3472, 20, 21.0–34.3 mm SL, municipality of Macaé, Rio de Janeiro State, Coastal Drainage.

*Lampiella gibbosa* (Miranda Ribeiro, 1908): LBP 2652, 8, 27.6–34.2 mm SL, municipality of Campinhos, Paraná State, rio Ribeira de Iguape.

*Microlepidogaster dimorpha* Martins & Langeani, 2011: LBP 10683, 2, 28.8–35.6 mm SL, municipality of Uberaba, Minas Gerais State, upper rio Paraná basin.

*Otothyris travassosi* Garavello, Britski & Schaefer, 1998: LBP 1971, 13, 14.0–27.2 mm SL, municipality of Canavieiras, Bahia State, Coastal Drainages.

*Otothyropsis marapoama* Ribeiro, Carvalho & Melo, 2005: LBP 4698, 6, 23.9–36.3 mm SL, municipality of Marapoama, São Paulo State, rio Tietê basin.

*Parotocinclus amazonensis* Garavello, 1977: MZUSP 10145, holotype, 14.6 mm SL, municipality of Coari, Amazonas State, rio Amazonas basin.

*Parotocinclus* cf. *bahiensis* (Miranda Ribeiro, 1918): LBP 7182, 3, 27.9–35.6 mm SL, municipality of Lençóis, Bahia State, Coastal Drainages.

*Parotocinclus longirostris* Garavello, 1988: MZUSP 36891, holotype, 27.8 mm SL, municipality of Manaus, Amazonas State, Amazon basin.

*Parotocinclus maculicauda* (Steindachner, 1877): LBP 2869, 15, 20.2–44.7 mm SL, municipality of Miracatu, São Paulo State, rio Ribeira de Iguape basin, LBP 3181, 1, 40.3 mm SL, municipality of Tapiraí, São Paulo State, rio Ribeira do Iguape basin.

*Parotocinclus prata* (Ribeiro, Melo & Pereira, 2002): MZUSP 68359, holotype, 37.5 mm SL, municipality of Presidente Olegário, Minas Gerais State, ribeirão Quiricó; LBP 11683, 3, 18.6–29.6 mm SL, municipality of Claro de Minas, Minas Gerais State, rio São Francisco.

*Pseudotothyris obtusa* (Miranda Ribeiro, 1911): LBP 898, 17, 23.6–30.9 mm SL, municipality of Cajati, São Paulo State, rio do Queimado.

*Schizolecis guntheri* (Miranda Ribeiro, 1918): LBP2123, 21, 28.4–36.3 mm SL, municipality of Parati, Rio de Janeiro State, Coastal Drainages, LBP 3546, 77, 20.9–35.8 mm SL, municipality of Ubatuba, São Paulo State, Coastal Drainages.

## Supplementary Material

XML Treatment for Parotocinclus dani
